# Epileptiform Activity in Alcohol Dependent Patients and Possibilities of Its Indirect Measurement

**DOI:** 10.1371/journal.pone.0018678

**Published:** 2011-04-26

**Authors:** Petr Bob, Denisa Jasova, Gustav Bizik, Jiri Raboch

**Affiliations:** Center for Neuropsychiatric Research of Traumatic Stress, Department of Psychiatry, First Faculty of Medicine, Charles University, Prague, Czech Republic; INSERM U901, France

## Abstract

**Background:**

Alcohol dependence during withdrawal and also in abstinent period in many cases is related to reduced inhibitory functions and kindling that may appear in the form of psychosensory symptoms similar to temporal lobe epilepsy frequently in conditions of normal EEG and without seizures. Because temporal lobe epileptic activity tend to spread between hemispheres, it is possible to suppose that measures reflecting interhemispheric information transfer such as electrodermal activity (EDA) might be related to the psychosensory symptoms.

**Methods and Findings:**

We have performed measurement of bilateral EDA, psychosensory symptoms (LSCL-33) and alcohol craving (ACQ) in 34 alcohol dependent patients and 32 healthy controls. The results in alcohol dependent patients show that during rest conditions the psychosensory symptoms (LSCL-33) are related to EDA transinformation (PTI) between left and right EDA records (Spearman r = 0.44, p<0.01).

**Conclusions:**

The result may present potentially useful clinical finding suggesting a possibility to indirectly assess epileptiform changes in alcohol dependent patients.

## Introduction

Alcohol dependence is characterized by an obsessive need for alcohol intake that is related to a subjective experience of craving that motivates to use alcohol and plays a significant role in maintenance of dependent behavior [1]–[3]. According to recent findings, alcohol dependence during alcohol withdrawal and also in patients who are in abstinent period may be related to sensitization and kindling [4], [5]. These processes may contribute to progressive changes reducing the threshold needed to trigger the risk of relapse, alcohol-related brain damage, cognitive impairment and may cause neurobehavioral alterations that may affect craving and other psychopathological symptoms [1]–[5]. There is also evidence that kindling causes abnormalities in a number of neurotransmitter systems and is related to reduced inhibitory functions, and increased activity of excitatory systems [1], [2] similar to human limbic or temporal lobe epilepsy [6]–[8]. These findings are in agreement with evidence that alcohol facilitates inhibitory gamma-aminobutyric acid (GABA) function and alcohol dependence is associated with time-dependent changes in brain GABA(A) receptor density and subunit gene expression levels that contribute to an understanding of the role of GABA systems in alcohol action and dependence, and the vulnerability to alcoholism [9], [10]. In this context, there is also evidence that many alcohol dependent patients have positive clinical response to anticonvulsant treatment although further research is still needed to identify which subtypes of alcoholic patients may benefitt from a particular type of medication [11], [12].

Together these findings suggest that in many cases alcohol dependence and its pathogenesis may be closely related to an epileptiform process most likely located in temporolimbic structures that are functionally related to cognitive, affective and memory processes and also involved in generation of temporal lobe seizures that may occur without any evidence on scalp EEG [13]–[16]. Inhibitory deficits and increased activity of excitatory systems in these brain areas has been called limbic irritability [17] and its symptoms may emerge in the form of psychosensory symptoms related to cognitive, affective, memory, sensory, behavioral and somatic disturbances similar to several symptoms of temporal lobe epilepsy [13]–[15], [18].

Although seizures in temporal lobe structures may occur only unilaterally, frequently the seizure activity tend to extend to the other hemisphere which significantly influences interhemispheric information transfer that may reflect temporal lobe “epileptogenicity” [19], [20]. These findings suggest that increased interhemispheric information transfer may present physiological indicator that could distinguish between alcohol dependent patients who frequently experience the symptoms of limbic irritability in comparison to them who experience these symptoms only rarely. Because alcohol dependent patients predominantly have normal EEG without epileptiform abnormalities and conventional scalp EEG is not able to provide information on subcortical structures, it is likely that direct measurement of limbic irritability and its manifestations using EEG likely is not possible. Although some new methods of EEG analysis potentially could help to detect limbic seizures with relatively new techniques that involve source analysis, coherence or using surface measures pointing to focal deregulation [21], [22], currently there is no evidence of reliable EEG indices for subcortical epileptiform discharges.

Nevertheless there is evidence that epileptiform activity may manifest in the autonomic nervous system [23], [24] which suggests a possibility that subcortical discharges could be reflected through its influences on the autonomic nervous system. With respect to these data recent evidence indicates that sensitive measure of autonomic changes reflecting limbic functions presents bilateral electrodermal activity (EDA). The evidence shows that EDA is governed by ipsilateral limbic modulation influences and correlates with amygdala activity, although also other structures, such as the ventromedial and dorsolateral prefrontal cortices, anterior cingulate gyrus, parietal lobe, insula, and hippocampus are also involved in EDA modulation [25], [26]. Evidence for the role of the amygdala in the expression of EDA mainly comes from functional-imaging studies and lesion studies [26]. Further evidence provides intracranial data by Mangina and Beuzeron-Mangina [25], who reported that rapid and discontinuous changes in limbic EEG activity induced by electric stimulation are linked to rapid and discontinuous changes in EDA, which suggests a direct functional connection between limbic EEG activity and changes in EDA. This finding suggests a hypothesis that EDA could reflect increased limbic excitability or irritability that is not directly presented on scalp EEG. In this context, it is possible to suppose that bilateral EDA could reflect interhemispheric information transfer (transinformation) related to extension of epileptiform activity between left and right temporal lobe structures during resting conditions detected in EDA baseline activity that is not disturbed by outside stimuli.

## Methods

### Participants

For empirical examination of the suggested hypothesis, methods of psychometric measures were used in 34 alcohol dependent outpatients of a university outpatient center (mean age: 38.42, age range: 31–50, SD = 4.83 years; 20 males and 14 females). The patients had a diagnosis of alcohol dependence with abstinence periods of one to six months from the beginning of alcohol withdrawal and treatment. The majority of the patients manifested comorbidities, i.e. anxiety (N = 5), affective disorders (N = 9), or personality disorders (N = 16) confirmed by clinical interview according to DSM IV criteria [27] and were also assessed by structured psychiatric interview M.I.N.I. version 5.0.0 [28]. Twenty-two patients of the sample were also smokers. The patients' treatment at the time of recruitment was based only on anxiolytic or antidepressant medication and disulfiram. Disulfiram treatment was used in 21 patients of the sample, with the beginning of disulfiram therapy approximately four weeks after the beginning of alcohol withdrawal. Anamnestic data showed that 18 patients had a history of detoxification treatment (mean number of detoxification treatments for the whole group was 1.49, SD = 1.41), but only one patients also had a history of alcohol withdrawal seizures. Exclusion criteria were alcohol addiction lasting more than 15 years, alcohol dementia, current alcohol withdrawal seizures and actual detoxification therapy, acute stage of alcohol withdrawal with abstinence symptoms, treatment lasting more than 6 months from the beginning of alcohol withdrawal, drug abuse, organic illnesses involving the central nervous system, psychotic disorders, electroconvulsive therapy, any form of epilepsy and epileptiform EEG changes, and mental retardation (IQ Raven higher than 90). The same exclusion criteria regarding CNS disorders were applied to the healthy controls together with the criterion that all the controls must be psychiatrically healthy and must be free of alcohol and other drug abuse according to M.I.N.I. The control group included 32 healthy participants (mean of age 36.81, age range 20–47, SD = 9.22; 18 men and 14 women). The healthy controls were recruited from general population by advertising and included hospital and university stuff members (N = 20) and university students (N = 12). Both the patients and the control group involved participants predominantly with highschool education. All the patients and controls gave written informed consent and the clinical study was approved by the the ethical committee of the First Faculty of Medicine of the Charles University in Prague.

### Psychometric measures

For the assessment of alcohol craving the revised version of the alcohol craving questionnaire (ACQ-R) was used [29]. The ACQ-R is a 30-item questionnaire for assessing craving that reflects the main dimensions of alcohol craving, such as urges and desires to use alcohol, intent to use alcohol, anticipation of positive outcome, anticipation of relief from withdrawal or negative outcome, and a lack of control over alcohol consumption [29]. Subjects indicate the degree of their experience on a seven-point Likert scale (from strongly agree to strongly disagree). The ACQ shows well psychometric properties and internal consistency (Cronbach's alpha 0.93).

Symptoms of limbic irritability linked to temporal lobe epileptiform activity were assessed by the Limbic System Checklist, LSCL-33 [17]. LSCL-33 is designed to measure temporo-limbic activity in the form of somatic, sensory, behavioral, and memory symptoms known to be associated with phenomena of ictal temporal lobe epilepsy. LSCL-33 shows well psychometric properties and internal consistency (Cronbach's alpha 0.90). These symptoms may be generally described as brief hallucinations, paroxysmal somatic disturbances, automatisms, and dissociative disturbances. Subjects indicate the degree of their experience on a four-point Likert scale (never, rarely, sometimes, often). The psychometric measures were administered individually in a quiet room with the help of a physician.

### EDA measurement

EDA was recorded bilaterally using a two-channel SAM unit and Psylab software (Contact Precision Instruments) connected to a personal computer with sampling frequency 1000 Hz. The measurements were performed in a quiet room with a room temperature of about 23°C. During the measurement the participant was in resting state and sat in a comfortable chair. The measurement was performed using two pairs of Ag/AgCl electrodes (8 mm diameter active area) filled with electroconductive paste that were attached to the medial phalanges of the index and middle finger of each hand.

### Data analysis

Because observational data reflect only a few real independent variables of a system, useful approaches to studying complex dynamical systems, such as the human brain, present methods of time-series analysis [30]. Approximation of the dynamic system behavior uses a finite number of (mathematically reconstructed) variables to approximate states of the system or relationships between the subsystems. Data for this analysis may provide, for example, a psychophysiological measurement performed on the system during an experiment. In this context it is possible to use a measure of the mutual interaction and information flow between subsystems that may be computed in the phase space using coupling measures such as pointwise transinformation (PTI). This coupling measure takes into account also nonlinear dependencies and can be applied to nonstationary time series. The PTI of two observable quantities has been derived from Shannon's information concept and is calculated from the probability densities of the observables in the phase space [31]. The method may be applied to the recorded signals representing the complex couplings of the physiological subsystems (in this case left and right side of the EDA).

This method of nonlinear data analysis was applied to 100 seconds long left- and right- EDA time series during rest using algorithm for pointwise transinformation which is included in software package Dataplore. The algorithm is performed through calculation of transinformation between EDA time series ×1 (left) and ×2 (right). The pointwise transinformation is a time-resolving variant of the transinformation, returning an estimation of the transinformation for each sample (point in time).

The defining formula for the i-th time step is:
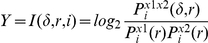
where the transinformation I is a function of the relative shift and relative phase space radius r, given for every sample point i. It denotes the probability to find a point of the reconstructed trajectory within a sphere of radius r around the i-th phase space point and refers to the phase spaces of the time series ×1 and ×2.

Statistical evaluation of PTI values (in bits), ACQ and LSCL-33 values was performed using software package Statistica version 8.0 and included descriptive statistics, Spearman correlations, multiple linear regression analysis and nonparametric Mann-Whitney test for independent samples.

## Results

The results show that during rest conditions the patients with higher level of symptoms of limbic irritability (LSCL-33) display increased level of interhemispheric information transmission measured by PTI calculated between left and right EDA records in comparison to patients who have lower LSCL-33 score (Table 1).

**Table 1:** Between group comparison for alcohol dependent patients with higher and lower level of symptoms related to limbic irritability (LSCL-33).

	Mean highLSCL-33±S.D.	Mean lowLSCL-33±S.D.	MW-testZ	P
**ACQ**	41.94±14.06	31.00±13.73	1.911	0.055
**LSCL-33**	52.11±13.08	21.05±8.99	4.977	<0.001
**PTI**	1.74±0.47	1.25±0.47	3.013	0.002

***Note.*** LSCL-33- Limbic System Checklist; ACQ- Alcohol Craving Questionnaire; PTI- information flow (pointwise transinformation in bits); Higher LSCL-33 (N = 17, LSCL-33≥36); Lower LSCL-33 (N = 17, LSCL-33<36); df = 32.

**Figure 1:**
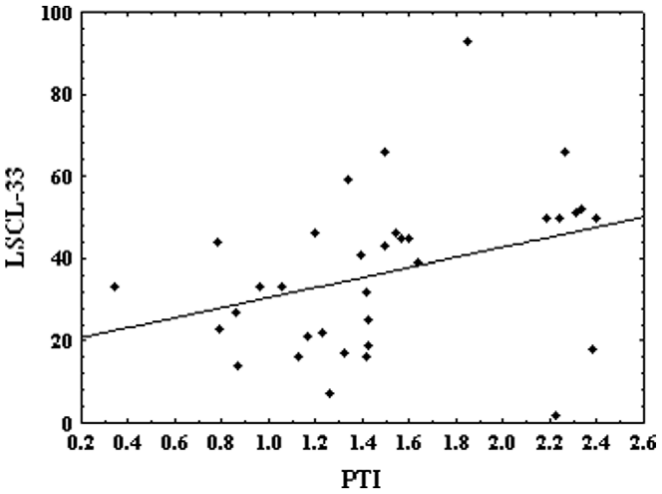
Dependency graph between pointwise transinformation- PTI (in bits) and symptoms of limbic irritability- LSCL-33 (r = 0.44, p<0.01).

The relationship between symptoms of limbic irritability and interhemispheric information transfer also reflects significant Spearman correlation between LSCL-33 and PTI (r = 0.44, p = 0.008) (Figure 1). Significant correlation has been found also between ACQ and LSCL-33 (r = 0.51, p = 0.002). Correlation between PTI and ACQ (r = 0.06, p = 0.71) was not statistically significant.

To distinguish the effect of ACQ and PTI on LSCL-33, we have used a multiple linear regression because it may be useful to know whether alcohol craving and PTI in their specific interactions are linked to increased levels of LSCL-33 and whether ACQ and PTI are tightly linked together. The result shows that multiple R = 0.72 is statistically significant (p = 0.000013; F = 16.58295) which enables to define LSCL-33 as a linear function of two variables LSCL-33 = *f* (ACQ, PTI).

In further evaluation we have also performed correlational analysis of the psychometric measures with EDA activity (skin conductance level) for each patient on both hands. The results indicate that there is no direct association between EDA and psychometric measures. Anamnestic data regarding the history of detoxification treatment did not show any significant relationships with limbic irritability and alcohol craving. Also we have not found significant differences related to age, sex and education.

We have also tried to find whether a subgroup of patients in the total group with a very strong relation of PTI to LSCL-33 is distinguished in some variables in comparison to the others. With this aim we have compared patients who have both PTI and LSCL-33 higher or lower than median with the others and did not find significant differences between the subgroups.

The same correlational analysis of the psychometric measures with PTI and EDA activity (skin conductance level) for each participant on both hands we have also performed in the healthy control group. The results indicate that there is no direct association between PTI, EDA and LSCL-33.

## Discussion

The results indicate that transinformation (PTI) between two EDA channels is related to symptoms of limbic irritability but not to symptoms of alcohol craving. The transinformation may capture information flow between two EDA channels but it may reflect also a simultaneous increase of EDA due to synchronous increase in limbic irritability on both sides. With respect to current findings this process could be explained as a consequence of latent temporal lobe epileptiform process with manifestations suggesting focal seizures and kindling related to alcohol dependence in structures related to cognitive, memory and.emotional processing.

In this context, the hypothesis that increased EDA transinformation may be explained by covered epileptic-like process seems to be relevant, but this description in principle does not mean that these patients have underlying epilepsy. With respect to close association of LSCL-33 symtoms with craving (ACQ) and PTI, it is likely that some symptoms of LSCL-33 could reflect an increased emotional tension related to craving at least in a subgroup of alcohol dependent patients. Although the symptoms of limbic irritability in several alcohol dependent patients may be linked to cerebral damage, head injury, infection and other factors, it is also possible that these symptoms might be influenced by cognitive and emotional changes related to alcohol craving. These changes may be related to deficits in inhibitory functions and increased excitability of the limbic system. Taken together these clinical findings are in agreement with evidence that focal seizures restricted to the hippocampus may progress without neurological clinical symptoms that are manifested when the focal hippocampal seizures spread to other structures such as the parahippocampal cortices and amygdala [16].

The relationship between EDA transinformation and symptoms of limbic irritability is also in agreement with findings suggesting that an important factor that implicates transition of latent epileptiform process to clinical seizure is the speed of interhemispheric propagation of established epileptiform activity [19], [20]. This process likely is related to changes in interhemispheric information transfer that might be linked to latent epileptiform process which may be regulated by interhemispheric inhibitory mechanisms. These regulatory mechanisms enable to block the clinical seizures and may reduce it to indirect clinical manifestations such as various forms of cognitive, affective, memory, sensory behavioral and somatic symptoms without full manifestations of epileptic clinical symptoms [14], [16], [18]. Within this context relationship between seizure-like symptoms of limbic irritability and EDA transinformation presents potentially useful clinical finding which could indirectly indicate spread of epileptiform activity between hemispheres that increases interhemispheric information transfer. Because the data acquisition and analysis are not time consuming the results potentially could present a helpful criterion for indication of anticonvulsant treatment which might be useful for clinical decision in alcohol dependent patients.
